# Comparative efficacy and safety of traditional Chinese patent medicine for anxiety disorders in children or adolescence: A protocol for systematic review and network meta-analysis

**DOI:** 10.1097/MD.0000000000022274

**Published:** 2020-09-25

**Authors:** Zhenyuan Jiang, Jiahao Wang, Xiaowen Yu, Chuancheng Li, Yuze Shao, Zhonglin Wang

**Affiliations:** a First College of Clinical Medicine, Shandong University of Traditional Chinese Medicine; b Affiliated Hospital of Shandong University of Traditional Chinese Medicine, Jinan, Shandong Province, China.

**Keywords:** anxiety disorders in childhood or adolescence, network meta-analysis, protocol, traditional Chinese patent medicine (TCPM)

## Abstract

**Background:**

Anxiety is the most common mental illness among adolescents and children, and its incidence is increasing year by year, which has a serious adverse effect on the academic and growth of adolescents and children. Conventional treatment methods such as oral administration of western medicine and psycho-behavioral therapy have obvious limitations. Chinese patent medicines play an irreplaceable role in the treatment of this disease. At present, there is no comparison of the safety and effectiveness of various Chinese patent medicines curing anxiety in adolescents. So we take advantage of the method of network meta-analysis to systematically compare the efficacy of various Chinese patent medicines curing this disease.

**Methods:**

We will systematically and comprehensively search the following databases, including PubMed, Web of Science, EMBASE, The Cochrane Library, China BioMedical Literature (CBM), China National Knowledge Infrastructure (CNKI), Chinese Scientific Journals Database (VIP), and Wanfang database. We will include all RCT trials that meet the inclusion criteria, starting from the establishment of the database until August 2020. Two researchers will independently screen the literature based on inclusion criteria. While extracting data, we also assess the risk of bias in the included studies. All the data and evidence obtained will be evaluated by the method of Bayesian network meta-analysis. STATA and WinBUGS software will be used.

**Results:**

This study will evaluate the effectiveness and safety of various TCPMs for anxiety disorders in children or adolescence.

**Conclusion:**

The results of this study will provide valuable references for the clinical application of Traditional Chinese patent medicines, and assist clinicians in formulating more reasonable diagnosis and treatment strategies.

**Ethics and dissemination:**

This study does not require ethical approval.

**INPLASY registration number:**

INPLASY202080048.

## 1 Introduction

Anxiety is the appearance of inner fear and restlessness for no obvious reason, often accompanied by autonomic dysfunction, and the clinical symptoms are persistent mental stress or episodic panic.^[1–4]^ Adolescents and children are in a special period of physical and mental development. Due to their young age and poor mental endurance, they are vulnerable to adverse life events,^[5]^ Therefore, Anxiety symptoms are very common especially in adolescents and children. Relevant research shows that about 5% to 20% of children and adolescents worldwide have anxiety disorders.^[6]^ A Norwegian survey on the stability, changes, and incidence of anxiety symptom clusters among 13 to 16-year-olds found that the incidence of high-level anxiety was 8.2%.^[7]^ Children suffering from anxiety disorders are vulnerable to other mental problems and related physical symptoms in the later growth process than other children.^[8–11]^

The cause of this disease is very complex and closely related to genetic factors,^[12,13]^ researches have confirmed that if 1 parent suffering from anxiety disorder, the risk of their children suffering from an anxiety disorder is significantly increasing; When both parents suffer from anxiety and other similar mental illnesses, the risk of children suffering from this illness is particularly high.^[14]^ Inappropriate parental education methods, such as excessive protection or excessive restraint, can increase the risk of social phobia in their children; Parents indifference and tyranny towards their children increase the risk of almost all diseases, but it is more closely related to anxiety and other mental disorders. Childrens excessive attachment to their parents is also a significant cause of this disease.^[15]^ Also, excessive study pressure, campus bullying, and strained relationships with classmates are also risk factors for this disease. This disease has a significant impact on the mental and social life of adolescents, severely affects learning efficiency, reduces social skills, and makes it hard to integrate into social life as an adult.^[16]^ In addition, due to the invisibility of the disease, it brings a great economic burden to patients and the medical system. According to estimates by Chisholm et al, anxiety and depression cost the world US$925 billion in productivity losses each year.^[17]^

Although a lot of research has been conducted, the pathogenesis of anxiety disorder is still not very clear, involving the regulation of multiple system functions. Neurotransmitters distributed in the limbic system, hippocampus, raphe nucleus and nucleus septum, such as serotonin (5-HT), dopamine (DA), and norepinephrine (NE), are closely related to the regulation of emotions. A large number of basic experiments have shown that changes in its concentration can cause mental disorders,^[18–20]^ and anxiety is one of the most common. Changes in the quantity and sensitivity of NMDA receptors in the brain can also cause anxiety symptoms.^[21]^ Immune dysfunction is another important mechanism that causes anxiety symptoms; Studies have shown that the dysfunction of microglia and CD4^+^ cells can significantly increase the anxiety behavior of experimental animals. In addition, oxidative stress and endocrine disorders in the brain are also critical mechanisms for anxiety disorders.^[22]^ According to the symptoms and severity of the disease, there are many treatments for anxiety disorders in adolescents and children. The drugs currently used to treat anxiety disorders mainly include weak tranquilizers (anxiolytics) and antidepressants. Benzodiazepines are widely used anti-anxiety agents, but their effects are short-lived, and they have side effects such as drowsiness, ataxia, and respiratory depression, so they are not suitable for long-term use. Commonly used antidepressants such as paroxetine and escitalopram have the dual effects of anti-anxiety and anti-depression, but their consequences are relatively slow and require long-term use. However, the nervous system of adolescents has not fully developed and matured. Animal experiments have shown that the results of SSRIs on adult and adolescent neurons are different. Exposure to psychiatric drugs during brain development may cause unpredictable short-term and long-lasting neuronal outcomes. Such medicine should be used with caution.^[23–25]^ Cognitive-behavioral-therapy (CBT) is currently the recommended first-line evidence-based treatment for anxiety disorders in children and adolescents.^[26]^ Comparing the CBT treatment group with no treatment group, the remission rate is about 60%. This result indicates that 40% of adolescents suffering from anxiety disorders still have no effect on CBT treatment.^[27]^ Chinese patent medicine (TCPM) is a powerful weapon of traditional Chinese medicine to cure diseases. It plays a significant role in relieving clinical symptoms, improving disease prognosis, and preventing recurrence. After long-term use and clinical observation, traditional Chinese patent medicine have the advantages of small side effects, high safety, and reliable efficacy. Many clinical trials and systematic reviews have confirmed the clinical efficacy of TCPM in the treatment of anxiety. Traditional Chinese patent medicines which are wildly used for treating anxiety include Xiaoyao Pills, Wuling Capsules, Shugan Jieyu Capsules, and Xuefu Zhuyu Oral Liquid. Basic research has shown that Xiaoyao Pill can reduce the expression of NSF and PICK1 protein by increasing GluR2/3 in the brain, inhibiting neuroinflammation induced by lipopolysaccharide, and alleviating anxiety symptoms.^[28,29]^ Xuefu Zhuyu Oral Liquid can improve the mental symptoms of model rats by increasing the content of monoamine neurotransmitter 5-HT and BDNF.^[30]^ Network meta-analysis can compare the advantages and disadvantages of 3 or more treatment methods, making full use of clinical data than traditional meta-analysis. Therefore, we use it to compare the effectiveness and safety of each traditional Chinese patent medicine in treating anxiety disorders in adolescents and children

## 2 Methods

We will use Bayesian NMA. Then we compliant PRISMA-P guidelines to conduct this study.

### 2.1 Study registration

This NMA has been registered on the International Platform of Registered Systematic Review and Meta-analysis Protocols (INPLASY) and the registration number is INPLASY202080048 (URL = https://inplasy.com/inplasy-2020-8-0048/).

### 2.2 Inclusion criteria

#### 2.2.1 Type of study

We will include all RCTs using TCPM for the treatment of anxiety in adolescents and children, as well as related clinical trials, such as I/II early stage, stage III trials, prospective and retrospective observational studies. The language is limited to Chinese and English.

#### 2.2.2 Participants

Adolescents and children diagnosed with anxiety disorders will be included. The diagnosis of anxiety will follow the Hamilton Anxiety Scale (HAMA) and Anxiety Self-Rating Scale (SAS).

#### 2.2.3 Interventions

The experimental group was treated with traditional Chinese patent medicines combined with conventional Western medicine. Chinese patent medicines included Xiaoyao Pills, Wuling Capsules, Shugan Jieyu Capsules, and Xuefu Zhuyu Oral Liquid. The control group received Western medicine treatment, including oral medication or mental behavior therapy. RCTs that use 2 or more proprietary Chinese medicines or combined acupuncture, moxibustion, and other traditional Chinese medicine methods are excluded.

#### 2.2.4 Outcomes

According to the Hamilton Anxiety Scale, a 5-point scale of 0–is adopted. The main indicators are: total clinical effective rate, improvement of anxiety mood, insomnia remission rate, improvement of cognitive function; secondary indicators including relapse rate, plant Nervous system symptoms and the rate of improvement in behavior when talking to people. The included literature must cover 1 or more main indicators.

### 2.3 Database and search strategy

We will search the Cochrane Library, PubMed, Embase, Clinical Trials, CNKI Database, VIP, Wanfang Database, and China Biomedical Database. The search strategy will be constructed in the form of Medical Subject Headings (MeSH) combine with keywords, including “Traditional Chinese patent medicine, TCPM, Anxiety disorders in children or adolescence, Adolescent generalized anxiety, Panic Disorder in childhood or adolescence, social phobia in children or adolescence, Randomized controlled,” etc. The search time limit is from the establishment of each database to August 2020. (The retrieval scheme of the PubMed database is listed in Table 1.)

**Table 1:**
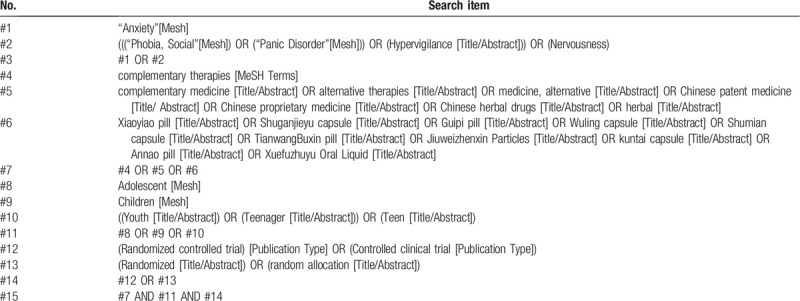
Detailed search strategy for PubMed.

### 2.4 Study selection and data extraction

The literature will be screened by 2 independent researchers according to the inclusion and exclusion criteria, and cross-checked them. In case of disagreement, they will discuss and negotiate with the third investigator. The extracted data include: ① the fundamental information of the included study (research title, first author, sample size, age, year, course of disease, treatment period); ② baseline characteristics and intervention measures of the research object; ③ key elements of bias risk evaluation; ④ outcome indicators.

### 2.5 Risk of bias assessment

The quality of each trial will be assessed by 2 researchers independently based on the Cochrane Risk of Bias Risk Assessment Tool recommended by Cochrane Handbook version 5.1.0. Use the decision words “high risk”, “low risk”, and “unclear risk” to evaluate the quality of the input article in 7 aspects, including: whether the random sequence is sufficient; whether there is hidden allocation; whether blinding is used; whether the result data is complete; whether there is selective reporting; whether there is publication bias; others.

### 2.6 Statistical analysis

We will use Stata 14.0 software and Markov chain-Monte Carlo (MCMC) method to conduct Bayesian meta-analysis. Three Markov chains will be used for simulation, and the number of iterations will be set at 50,000 (the first 20,000 are used for annealing to eliminate the effect of the initial value, and the last 30,000 are used for sampling).

The reticular diagram will be drawn by Stata 15.0 software to show the direct and indirect comparison between different interventions. The relative odds ratio (RoR) and its 95% confidence interval (CI) are calculated to evaluate the consistency of each closed loop. The lower limit of 95% CI is equal to 1, indicating good consistency. If RoR is close to 1, direct evidence and indirect evidence are consistent, and the fixed effect model is adopted for analysis. Otherwise, the closed-loop is considered to have obvious inconsistencies, and the random effect model is used for analysis. Dichotomous data will be represented by odds ratio (OR) and 95% CI, and *P* < .05 was considered statistically significant. WinBUGS 1.4.3 will be used to rank the efficacy of different interventions and the area under the curve will be recorded (the area under the curve will be expressed as a percentage, the larger the value, the better the effect).

### 2.7 Assessment of heterogeneity

If (*P* > .10 and *I*^2^ < 50%), we will use the fixed-effect model. Otherwise, we will further explore the source of heterogeneity, and if the source cannot be found, the random-effects model will be used for analysis.

### 2.8 Subgroup analysis and sensitivity analysis

Subgroup analysis will be considered if sufficient data is available. Sensitivity analysis will be conducted with symptom improvement rate to evaluate clinical similarity and methodology of included studies to determine the reliability of the results of this study.

### 2.9 Evaluation of publication bias

Total effective rate, anxiety after treatment, degree of nervousness after treatment, and sleep status after treatment will be taken as indicators, and the inverted funnel plot will be drawn with each effect amount as the horizontal coordinate and the standard error of effect amount as the vertical coordinate. If the inverted funnel plot is symmetric, it suggests that there is a small sample effect or a slight possibility of publication bias in this study.

### 2.10 Grading the quality of evidence

We will use GRADE to evaluate the quality of evidence from the following 5 aspects: risk of bias, indirectness, inconsistency, imprecision, and publication bias.^[31]^

## 3 Discussion

Teenagers are not fully mature mentally and are vulnerable to adverse life events. Mental health problems are common among children and adolescents, and anxiety disorder is the most common mental illness. Failure to receive timely and effective treatment will result in childrens academic frustration, substance abuse, and poor social function, which will seriously affect personal development. Whether it is cognitive-behavioral-therapy (CBT) or oral psychiatric drugs, there are obvious limitations. Traditional Chinese patent medicine are under the strict supervision of relevant national departments, according to traditional Chinese medicine theories, the prescriptions are selected with rigorous compatibility, and have been effective after long-term clinical application. The Chinese medicine decoction pieces are processed through scientific preparation techniques to produce pills, tablets, capsules, granules, and other different dosage forms are convenient to take, widely used, and have good therapeutic effects. At present, there is no comparison of the advantages and disadvantages of various Chinese patent medicines for the treatment of anxiety in adolescents, so it is necessary to use the method of network meta-analysis to study this topic. In this study, we will introduce the network meta-analysis on the basis of the existing RCT to evaluate the advantages and disadvantages of various Chinese patent medicines, so as to provide clinicians with more complete diagnosis and treatment plans.

## Author contributions

**Conceptualization:** Zhenyuan Jiang, Zhonglin Wang.

**Data curation:** Zhenyuan Jiang, Xiaowen Yu.

**Formal analysis:** Zhenyuan Jiang, Yuze Shao, Zhonglin Wang.

**Funding acquisition:** Zhonglin Wang.

**Investigation:** Zhenyuan Jiang.

**Methodology:** Zhenyuan Jiang, Jiahao Wang, Chuancheng Li.

**Project administration:** Zhenyuan Jiang.

**Resources:** Zhenyuan Jiang.

**Software:** Zhenyuan Jiang, Jiahao Wang, Xiaowen Yu.

**Validation:** Zhenyuan Jiang.

**Visualization:** Zhenyuan Jiang.

**Writing – original draft:** Zhenyuan Jiang, Jiahao Wang.

**Writing – review & editing:** Zhenyuan Jiang, Zhonglin Wang.
